# Influence of Delayed Conductance on Neuronal Synchronization

**DOI:** 10.3389/fphys.2020.01053

**Published:** 2020-09-03

**Authors:** Paulo R. Protachevicz, Fernando S. Borges, Kelly C. Iarosz, Murilo S. Baptista, Ewandson L. Lameu, Matheus Hansen, Iberê L. Caldas, José D. Szezech, Antonio M. Batista, Jürgen Kurths

**Affiliations:** ^1^Instituto de Física, Universidade de São Paulo, São Paulo, Brazil; ^2^Graduate Program in Science–Physics, State University of Ponta Grossa, Ponta Grossa, Brazil; ^3^Center for Mathematics, Computation, and Cognition, Federal University of ABC, São Paulo, Brazil; ^4^Faculdade de Telêmaco Borba, FATEB, Telêmaco Borba, Brazil; ^5^Graduate Program in Chemical Engineering, Federal Technological University of Paraná, Ponta Grossa, Brazil; ^6^Institute for Complex Systems and Mathematical Biology, SUPA, University of Aberdeen, Aberdeen, United Kingdom; ^7^Cell Biology and Anatomy Department, University of Calgary, Calgary, AB, Canada; ^8^Department of Mathematics and Statistics, State University of Ponta Grossa, Ponta Grossa, Brazil; ^9^Department of Physics, Humboldt University, Berlin, Germany; ^10^Department Complexity Science, Potsdam Institute for Climate Impact Research, Potsdam, Germany; ^11^Department of Human and Animal Physiology, Saratov State University, Saratov, Russia

**Keywords:** synchronization, integrate-and-fire, neuronal network, time delay, conductance

## Abstract

In the brain, the excitation-inhibition balance prevents abnormal synchronous behavior. However, known synaptic conductance intensity can be insufficient to account for the undesired synchronization. Due to this fact, we consider time delay in excitatory and inhibitory conductances and study its effect on the neuronal synchronization. In this work, we build a neuronal network composed of adaptive integrate-and-fire neurons coupled by means of delayed conductances. We observe that the time delay in the excitatory and inhibitory conductivities can alter both the state of the collective behavior (synchronous or desynchronous) and its type (spike or burst). For the weak coupling regime, we find that synchronization appears associated with neurons behaving with extremes highest and lowest mean firing frequency, in contrast to when desynchronization is present when neurons do not exhibit extreme values for the firing frequency. Synchronization can also be characterized by neurons presenting either the highest or the lowest levels in the mean synaptic current. For the strong coupling, synchronous burst activities can occur for delays in the inhibitory conductivity. For approximately equal-length delays in the excitatory and inhibitory conductances, desynchronous spikes activities are identified for both weak and strong coupling regimes. Therefore, our results show that not only the conductance intensity, but also short delays in the inhibitory conductance are relevant to avoid abnormal neuronal synchronization.

## 1. Introduction

Network physiology reveals how organ systems dynamically interact (Bartsch et al., 2015). The human organism is a complex physiological and integrated system in which a fail in a specific component can produce a range of biological effects (Bashan et al., 2012). One of the biggest challenges is to understand how global behavior of the human organism emerges due to local causes (Ivanov et al., 2016). Brain-brain and brain-organ networks have been considered to study integrated physiological systems under neuronal control (Ivanov et al., 2009). Chen et al. (2006) investigated the relationship between the blood flow velocities in the cerebral arteries and beat-to-beat blood pressure. Liu et al. (2015) built a network of brain wave interactions. They found complex brain dynamics, such as desynchronous and synchronous activities (Xu et al., 2006) during quiet wake and deep sleep, respectively.

Time delay has been considered in several problems of biological interest (Glass et al., 1988), such as herbivore dynamics (Sun et al., 2015), polymerization processes (Mier-y-Terán-Romero et al., 2010), dynamics of tumor growth (Byrne, 1997; Borges et al., 2014), and dynamic behavior of coupled neurons (Esfahani et al., 2016). One of the brain's intrinsic properties is the delay in the transmission of information among separate brain regions (Deco et al., 2009). Stoelzel et al. (2017) investigated the relation between axonal conduction delays and visual information. They found that some conduction times in corticothalamic axons exceed 50 ms. Conduction latencies in mammalian brain about 100 ms are also reported by Aston-Jones et al. (1985).

Dynamic brain behavior can be mimicked by means of neuronal network models (Protachevicz et al., 2019), for instance, neuronal synchronous behavior (Borges et al., 2018). Neuronal synchronization is found in task conditions (Deco et al., 2011). Furthermore, many neurological disorders are also related to synchronous behavior in the brain (Uhlhaas and Singer, 2006). Network models have been used to study the effects of time delay in synchronized neuronal activities (Stepan, 2009). Dhamala et al. (2004) showed the enhancement of neuronal synchrony by time delay in a neuronal network. Wang et al. (2016) investigated synchronized stability in coupled neurons with distributed and discrete delays. Kim and Lim (2018a,b) studied synchronization in networks, where they considered plasticity (Borges et al., 2017a) and time delays between the pre-synaptic and post-synaptic spike times.

Neurons can be modeled by differential equations. In 1907, Lapicque (Lapicque, 1907) used a linear differential equation (leaky integrate-and-fire) to simulate the neuron membrane potential. A system of non-linear differential equations was proposed by Hodgkin and Huxley (1952) to describe the action potential. The Hodgkin-Huxley model considers ion channels that open and close according to the voltage. Different connectivities among the neurons have been considered to form neuronal networks. The dynamics of coupled neurons was investigated in networks with random connections (Brunel, 2000), small-world (Tang et al., 2011), and scale-free (Batista et al., 2007, 2010) topologies were used to study neuronal synchronization.

We build here a network composed of adaptive exponential integrate-and-fire (AEIF) neurons. The AEIF model was introduced by Brette and Gerstner (2005). Depending on the parameter values, the AEIF neuron can exhibit different firing patterns (Naud et al., 2008). Synchronized firing patterns were observed in coupled AEIF neurons (Borges et al., 2017b). Pérez et al. (2011) studied the influence of conduction delays on spike synchronization in Hodgkin-Huxley neuronal networks. Previous works found that slow-rising inhibitory synaptic currents can induce synchrony (Abbott and van Vreeswijk, 1993; van Vreeswijk et al., 1994) and affect the stability of asynchronous state (e.g., splay state) (van Vreeswijk, 1996; Olmi et al., 2014). Chen et al. (2017) reported that the competition between coupling strength and synaptic time-constant leads to rich bifurcation in pulse-coupled neuronal networks with either excitatory or inhibitory synapses.

In this work, we study AEIF neurons randomly connected by means of excitatory and inhibitory conductivities. The neurons can exhibit not only spike but also burst activities (Santos et al., 2019). Our results show that the delayed conductance in both excitatory and inhibitory connections play an important role in the neuronal synchronization. Furthermore, we demonstrate that not only the values of the conductance intensity, but also small delays in inhibitory conductances are important to prevent abnormal synchronization.

The paper is organized as follows. In section 2, we introduce the neuronal network composed of AEIF neurons and delayed conductance. Section 3 shows our results about the effects of conduction delays in neuronal synchronization. We draw our conclusions in the last section.

## 2. Model and Methods

We construct a neuronal network with 100 AEIF neurons, where the connections are randomly chosen with probability equal to 0.5. The connection probability is defined as

(1) $$\begin{array}{l} p=\frac{N_{\text{T}}}{N\cdot \left(N-1\right)}, \end{array}$$

where *N*_T_ is the total connection number of the network and *N*·(*N* − 1) is the maximal possible number of connections for a network with *N* neurons without auto-connections. We consider that each neuron has at least one connection. The network has 80 and 20% of excitatory and inhibitory connections, respectively (Noback et al., 2005). The network dynamics is given by

(2) $$\begin{array}{l} C\frac{dV_{i}}{dt}=-g_{\text{L}}\left(V_{i}-E_{\text{L}}\right)+g_{\text{L}}\Delta_{\text{T}}\exp\left(\frac{V_{i}-V_{\text{T}}}{\Delta_{\text{T}}}\right)\\ \;-w_{i}+I_{i}+I_{i}^{\text{syn}}, \end{array}$$

(3) $$\begin{array}{l} \tau_{w}\frac{dw_{i}}{dt}=a_{i}\left(V_{i}-E_{\text{L}}\right)-w_{i}, \end{array}$$

(4) $$\begin{array}{l} \tau_{\text{s}}\frac{dg_{i}}{dt}=-g_{i}, \end{array}$$

where *V*_*i*_, *w*_*i*_, and *g*_*i*_ are the membrane potential, the adaptation current, and the conductance of the neuron *i*, respectively. We consider *C* = 200 pF (capacitance membrane), *g*_L_ = 12 nS (leak conductance), *E*_L_ = −70 mV (resting potential), *I*_*i*_ = 2·*I*_rheo_ (constant input equal to two times the rheobase current Naud et al., 2008), Δ_T_ = 2 mV (slope factor), *V*_T_ = −50 mV (potential threshold), and τ_*w*_ = 300 ms (adaptation time constant). The level of subthreshold adaptation *a*_*i*_ is randomly distributed in the interval [1.9, 2.1] nS. This set of parameters corresponds to the spike adaptation activity when neurons are uncoupled. In the model, the adaptation mechanism is able to generate burst activities when the neurons are connected by excitatory synapses (Fardet et al., 2018). For weak coupling, the neurons exhibit spike activities, while for strong, burst activities can occur for low inhibition (Protachevicz et al., 2019). The current input $I_{i}^{\text{syn}}$ is calculated by the expression

(5) $$\begin{array}{l} I_{i}^{\text{syn}}\left(t\right)=\overset{N}{\underset{j=1}{\sum }}\left[V_{\text{REV}}^{j}-V_{i}\left(t\right)\right]A_{ij}g_{j}\left(t-d_{j}\right), \end{array}$$

where *d*_*j*_ is the time delay in the conductance. We consider *d*_*j*_ = *d*_inh_ for inhibitory and *d*_*j*_ = *d*_exc_ for excitatory neurons. $V_{\text{REV}}^{j}$ is the reversal potential (*V*_REV_ = 0 mV for excitatory and *V*_REV_ = −80 mV for inhibitory synapses). In the adjacency matrix (*A*_*ij*_), the element value is equal to 1 when the presynaptic neuron *j* and post-synaptic neuron *i* are connected, and 0 when they are not connected. *g*_*j*_ has an exponential decay with the synaptic time constant τ_*s*_ = 2.728 ms. When the membrane potential of the neuron *i* is above a threshold (*V*_*i*_ > *V*_thres_) (Naud et al., 2008), the state variables are updated according to the rules

(6) $$\begin{array}{l} V_{i}\to V_{\text{r}},\\ w_{i}\to w_{i}+b,\\ g_{i}\to g_{i}+g_{s}, \end{array}$$

where *V*_r_ = −58 mV is the reset potential and *b* = 70 pA is the triggered adaptation addition. The chemical conductance *g*_s_ assumes *g*_exc_ and *g*_inh_ for excitatory and inhibitory neurons, respectively. We define a relative inhibitory conductance as *g* = *g*_inh_/*g*_exc_. Table 1 shows the standard parameter set that we use in our simulations.

**Table 1:** Standard parameter set.

**Parameter**	**Description**	**Value**
*N*	Number of AEIF on the network	100 neurons
*C*	Capacitance membrane	200 pF
*g*_L_	Leak conductance	12 nS
*E*_L_	Resting potential	−70 mV
*I*_*i*_	Constant input current	2·*I*_rheo_
Δ_T_	Slope factor	2 mV
*V*_T_	Potential threshold	−50 mV
τ_*w*_	Adaptation time constant	300 ms
*a*_*i*_	Level of subthreshold adaptation	[1.9, 2.1] nS
*b*	Level of triggered adaptation	70 pA
*V*_r_	Reset potential	−58 mV
$V_{\text{REV}}^{\text{exc}}$	Excitatory synaptic reversal potential	0 mV
$V_{\text{REV}}^{\text{inh}}$	Inhibitory synaptic reversal potential	−80 mV
*A*_*ij*_	Adjacent matrix elements	0 or 1
τ_*s*_	Synaptic time constant	2.728 ms
*t*_fin_	Final time to analyses	10 s
*t*_ini_	Initial time to analyses	5 s
*g*_s_	Chemical conductance	*g*_exc_ or *g*_inh_
*d*_*j*_	Time delay	*d*_exc_ or *d*_inh_

As a diagnostic tool to identify synchronization, we use the time average of the Kuramoto order parameter (Kuramoto, 1984; Batista et al., 2017)

(7) $$\begin{array}{l} \bar{R}=\frac{1}{t_{\text{fin}}-t_{\text{ini}}}\int_{t_{\text{ini}}}^{t_{\text{fin}}}|\frac{1}{N}\overset{N}{\underset{j=1}{\sum }}\text{exp}\left(\text{i}\Phi_{j}\left(t\right)\right)|dt, \end{array}$$

where the final time in the simulation and initial time for analyses are *t*_fin_ = 10 s and *t*_ini_ = 5 s, respectively. $\bar{R}$ ranges from 0 to 1 and approaches 1 for synchronous behavior. The phase of each neuron *j* is calculated by Pikovsky et al. (1997)

(8) $$\begin{array}{l} \Phi_{j}\left(t\right)=2\pi m+2\pi \frac{t-t_{j,m}}{t_{j,m+1}-t_{j,m}}, \end{array}$$

where *t*_*j,m*_ is the time at which neuron *j* suffers its *m*-th spike (*m* = 0, 1, 2, … ) and Φ is defined between two spikes in the interval [*t*_*j,m*_, *t*_*j,m*+1_].

The AEIF neuron can exhibit spike or burst activities. To identify these activities, we compute the coefficient of variation of the inter-spike interval (ISI)

(9) $$\begin{array}{l} \bar{\text{CV}}=\frac{\sigma_{\text{ISI}}}{\bar{\text{ISI}}}, \end{array}$$

where σ_ISI_ and $\bar{\text{ISI}}$ are the standard deviation and the mean value of ISI, respectively. We identify spike activities when $\bar{\text{CV}}<0.5$ and burst activities when $\bar{\text{CV}}\geq 0.5$ (Protachevicz et al., 2018).

We calculate the mean firing frequency $\bar{F}\left(\text{Hz}\right)$ of the neuronal network by mean of the expression

(10) $$\begin{array}{l} \bar{F}=\frac{1}{\bar{\text{ISI}}}. \end{array}$$

We also compute the instantaneous *I*^syn^(*t*) and the mean synaptic input ${\bar{I}}_{\text{s}}$ (pA) of the network through

(11) $$\begin{array}{l} I^{\text{syn}}\left(t\right)=\frac{1}{N}\overset{N}{\underset{i=1}{\sum }}I_{i}^{\text{syn}}\left(t\right), \end{array}$$

(12) $$\begin{array}{l} {\bar{I}}_{\text{s}}=\frac{1}{\left(t_{\text{fin}}-t_{\text{ini}}\right)}\int_{t_{\text{ini}}}^{t_{\text{fin}}}I^{\text{syn}}\left(t\right)dt, \end{array}$$

where $I_{i}^{\text{syn}}\left(t\right)$ is described by Equation (5). In all diagnostics, each point in the parameter space *d*_inh_ × *d*_exc_ is computed by means of the average of 10 different initial conditions. The initial conditions of *V*_*i*_ and *w*_*i*_ are randomly distributed in the interval *V*_*i*_ = [−70, −50] mV and *w*_*i*_ = [0, 80] nA, respectively. The initial conductance *g*_*i*_ is equal to 0 for all neurons. To solve the delayed differential equations, we consider an initial profile of the network (for *t* ∈ [−*d*_*j*_, 0]) in which the neurons are not spiking.

## 3. Results

**Figure 1:**
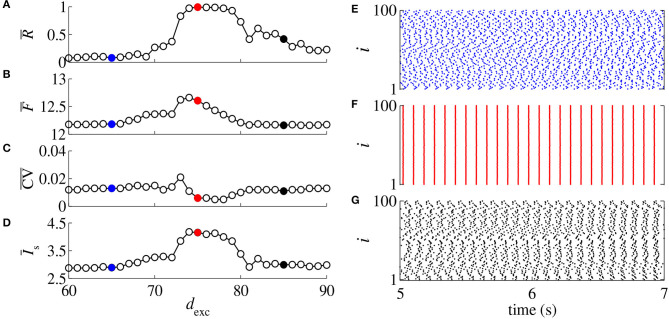
**(A)** Mean order parameter ($\bar{R}$), **(B)** mean firing frequency ($\bar{F}$), **(C)** mean coefficient of variation ($\bar{\text{CV}}$), and **(D)** mean synaptic input (${\bar{I}}_{\text{s}}$) as a function of the excitatory delayed conduction *d*_exc_. Raster plots for *d*_exc_ = 65 ms **(E)**, *d*_exc_ = 75 ms **(F)**, and *d*_exc_ = 85 ms **(G)** for *g*_exc_ = 0.2 nS, *g* = 6, and *d*_inh_ = 5 ms, and according to the colored circles.

Neuronal conductances play a key role in network responses to stimuli (di Volo et al., 2019). Conduction delays were observed between the activities of the pre-synaptic and post-synaptic neurons (Ermentrout and Kopell, 1998). Figures 1A–D display $\bar{R}$, $\bar{F}$, $\bar{\text{CV}}$, and ${\bar{I}}_{\text{s}}$, respectively, as a function of *d*_exc_ for *g*_exc_ = 0.2 nS, *g* = 6, and *d*_inh_ = 5 ms. In Figures 1E–G (blue points, red points, black points), we show the raster plots for the parameters indicated by the respective filled colored circles. In Figures 1A–D, increasing *d*_exc_ from 65 ms (blue) to 75 ms (red), the desynchronized spikes (Figure 1E) go to a synchronous behavior (Figure 1F), however, the spikes desynchronize when *d*_exc_ is increased to 85 ms (Figure 1G). We find that a small change of the delayed conductance value can improve or suppress synchronous behavior.

**Figure 2:**
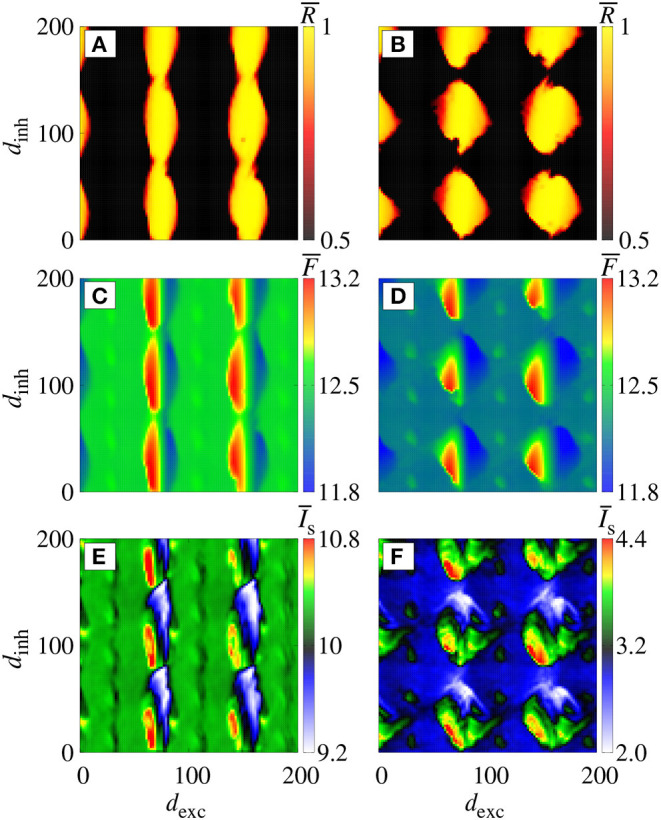
Colors represent $\bar{R}$, $\bar{F}$ and ${\bar{I}}_{\text{s}}$ on the parameter space *d*_exc_ × *d*_inh_ for *g*_exc_ = 0.2 nS, where we consider *g* = 2 in **(A,C,E)**, and *g* = 6 in **(B,D,F)**.

Figures 2A,B display the parameter space *d*_inh_ × *d*_exc_ for *g*_exc_ = 0.2 nS (weak coupling), where the color bar corresponds to the average order parameter $\bar{R}$. The parameter space exhibits synchronous (yellow region) and desynchronous (black region) spike patterns ($\bar{\text{CV}}<0.5$). For *g* = 2 (Figure 2A), we verify vertical domains of synchronization that can be reached by maintaining *d*_inh_ constant, and varying *d*_exc_. Increasing the relative inhibitory conductance for *g* = 6, separated domains with synchronized spikes appear, as shown in Figure 2B. For the considered parameter space, the highest and lowest values of $\bar{F}$ (Figures 2C,D) and $\bar{I_{\text{s}}}$ (Figures 2E,F) appear in the synchronized domain. In the domains with synchronized activities, we observe that the neuronal network achieves and maintains synchronized activities by means of changes in the mean firing frequency and synaptic current. In the region with a desynchronous pattern, the excitatory and inhibitory synaptic currents arrive in the neurons approximately at the same time.

**Figure 3:**
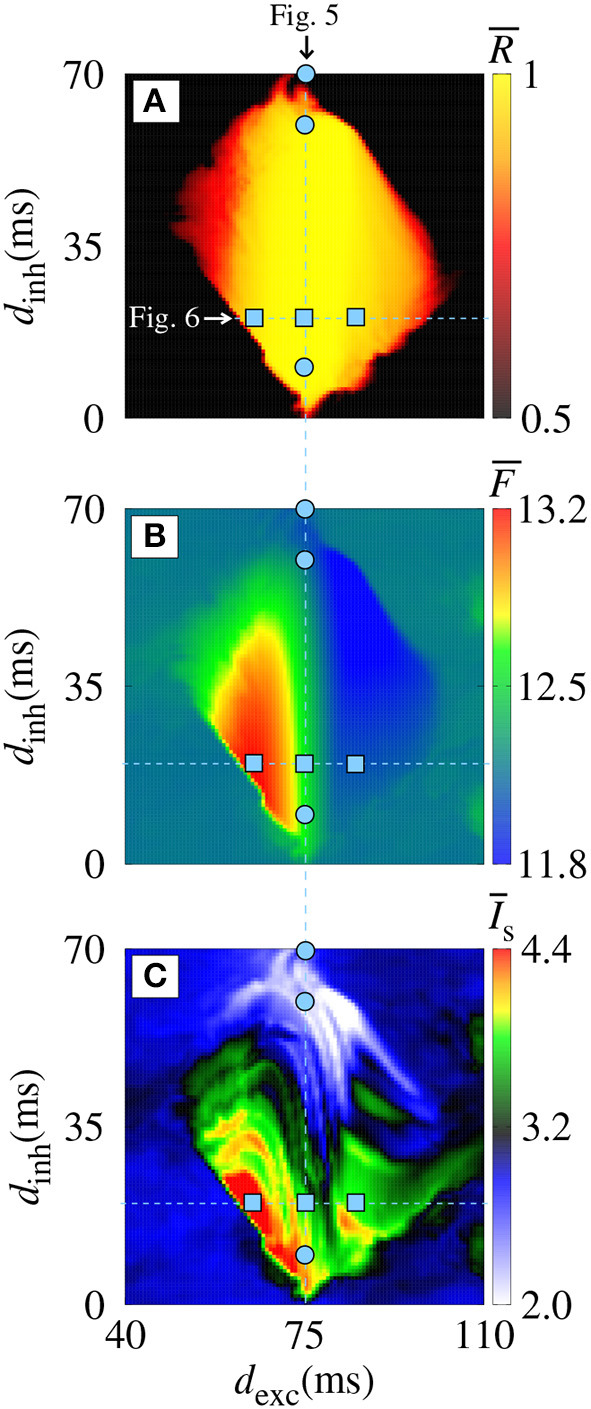
Magnifications of the parameter spaces shown in the right column of Figure 2. **(A)** $\bar{R}$, **(B)** $\bar{F}$, and **(C)** ${\bar{I}}_{\text{s}}$ for *g*_exc_ = 0.2 nS and *g* = 6 on the parameter space *d*_exc_ × *d*_inh_.

**Figure 4:**
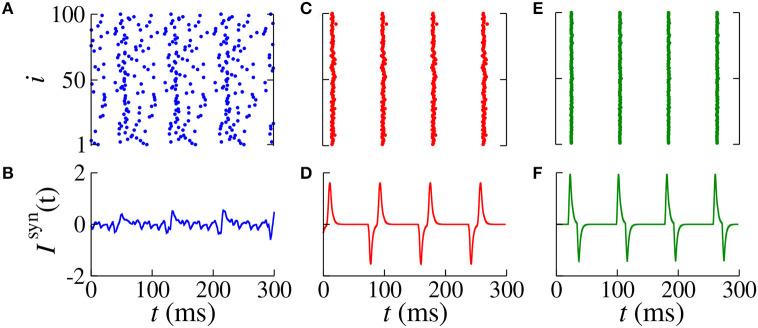
Raster plot (top) and *I*^syn^(*t*) (bottom) for *g*_exc_ = 0.2 nS, *g* = 6, and *d*_exc_ = 75 ms for different values of *d*_inh_ (blue circles in Figure 3). We consider **(A,B)** *d*_inh_ = 70 ms (blue), **(C,D)** *d*_inh_ = 60 ms (red), and **(E,F)** *d*_inh_ = 10 ms (green).

Figure 3 displays magnifications of the parameter spaces shown in the right column of Figure 2 (40 ≤ *d*_exc_ ≤ 110 ms and 0 ≤ *d*_inh_ ≤ 70 ms). In the domain with a synchronous pattern, we observe that *d*_exc_ and *d*_inh_ have a significant influence on the mean firing frequency and mean synaptic current, respectively. The dynamics of neurons for some values of *d*_exc_, indicated in the vertical line (blue circles) in Figure 3 for *d*_exc_ = 75 ms, are shown in Figure 4 by means of the temporal evolution of *i* (A,C,E) and *I*^syn^ (B,D,F), where we consider *d*_inh_ = 70 ms (blue), *d*_inh_ = 60 ms (red), and *d*_inh_ = 10 ms (green).

**Figure 5:**
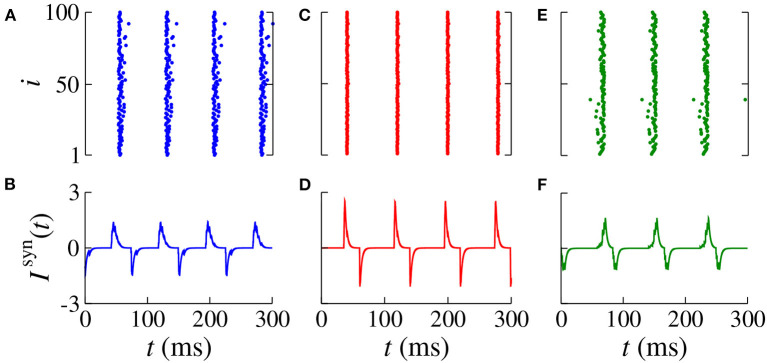
Raster plot (top) and *I*^syn^(*t*) (bottom) for *g*_exc_ = 0.2 nS, *g* = 6, and *d*_inh_ = 30 ms for different values of *d*_exc_ (blue squares in Figure 3). We consider **(A,B)** *d*_exc_ = 65 ms (blue), **(C,D)** *d*_exc_ = 75 ms (red), and **(E,F)** *d*_exc_ = 85 ms (green).

In Figures 4A,B, we verify the existence of desynchronous spikes when excitatory and inhibitory inputs arrive in almost the same time (*d*_exc_ ≈ *d*_inh_). Figure 5 shows raster plots (top) and *I*^syn^(*t*) (bottom) for *d*_inh_ = 30 ms (blue squares in Figure 3), where we consider *d*_exc_ = 65 ms (blue), *d*_exc_ = 75 ms (red), and *d*_exc_ = 85 ms (green). The parameters correspond to the region where synchronization can occur. Furthermore, we observe that depending on the excitatory delay value, synchronization can be improved. We verify that the synchronization is improved for *d*_exc_ = 75 ms, namely certain values of the delay can optimize the synchronization regime.

**Figure 6:**
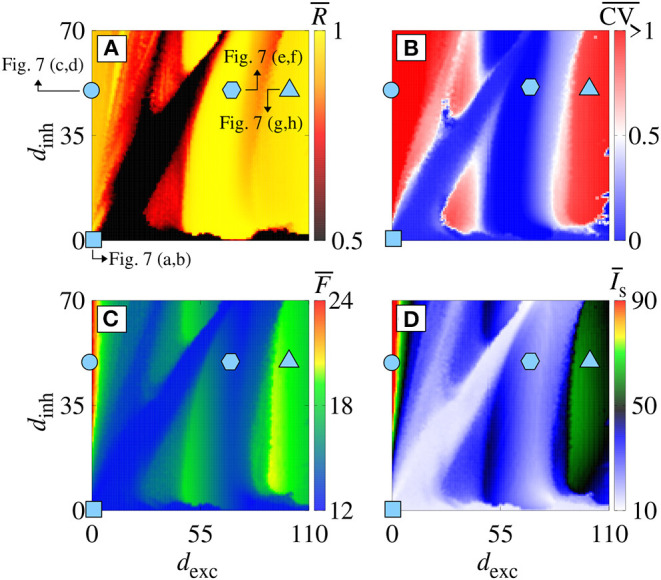
**(A)** $\bar{R}$, **(B)** $\bar{\text{CV}}$, **(C)** $\bar{F}$, and **(D)** ${\bar{I}}_{\text{s}}$ in the parameter space *d*_exc_ × *d*_inh_ for *g*_exc_ = 0.8 nS and *g* = 6. Symbols in *d*_exc_ × *d*_inh_ correspond to *d*_exc_ = *d*_inh_ = 0 ms (cyan square), *d*_exc_ = 0 ms and *d*_inh_ = 50 ms (cyan circle), *d*_exc_ = 70 ms and *d*_inh_ = 50 ms (cyan hexagon), and *d*_exc_ = 110 ms and *d*_inh_ = 50 ms (cyan triangle).

**Figure 7:**
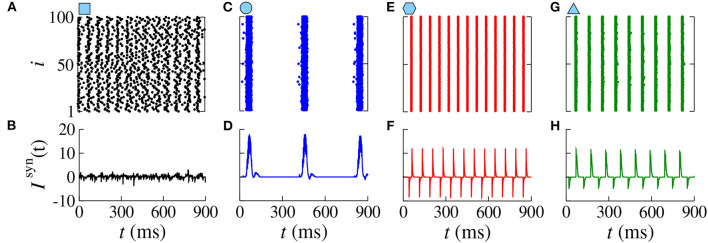
Raster plots (top) and *I*^syn^(*t*) (bottom) for *g*_exc_ = 0.8 nS, *g* = 6 for different values of *d*_exc_ and *d*_inh_. Different delay values generate desynchronized spikes **(A,B)**, synchronized bursts **(C,D)**, synchronized spikes **(E,F)**, and synchronized bursts **(G,H)**.

Increasing *g*_exc_ from 0.2 to 0.8 nS (strong coupling), in Figure 6, we observe in another range of the parameter space *d*_inh_ × *d*_exc_ (Figure 6A) where the region with synchronous behavior increases. Figure 6B displays the existence of regions with spike (blue) and burst (red) through the coefficient of variation value. Comparing Figures 6A,B, we verify that there are not only synchronized spikes, but also synchronized bursts. Moreover, desynchronous spike patterns are found for *d*_inh_ ≈ *d*_exc_. Figures 6C,D show in color scale the values of $\bar{F}$ and ${\bar{I}}_{\text{s}}$, respectively. We see that the synchronized spikes occur for the values of *d*_inh_ and *d*_exc_ in which $\bar{F}$ and ${\bar{I}}_{\text{s}}$ are low. The synchronized bursts can be found with different values of $\bar{F}$ and ${\bar{I}}_{\text{s}}$. In addition, we also observe desynchronized activities for *d*_inh_ ≈ *d*_exc_. Figure 7 displays the raster plot (top) and *I*^syn^(*t*) (bottom) for *g*_exc_ = 0.8 nS, *g* = 6, and different values of *d*_exc_ and *d*_inh_, according to the parameters pointed by the symbols in Figure 6. Different delay values can generate desynchronized spikes (Figures 7A,B), synchronized bursts (Figures 7C,D,G,H), and synchronized spikes (Figures 7E,F).

## 4. Discussion and Conclusion

In this paper, we investigate the influence of delayed conductance on the neuronal synchronization. The study of neuronal synchronization is of great importance in neuroscience, due to the fact that it has been related to cognition, as well as to brain pathology. The conductance between the neurons plays a crucial role in the synchronous behavior. Many studies investigated the effects of the conductance on the neuronal activities (Bezanilla, 2008; Kispersky et al., 2012).

We construct a network composed of adaptive exponential integrate-and-fire (AEIF) neurons. The AEIF neuron has been used to mimic spike and burst patterns. In our network, we consider that the neurons are randomly connected by means of inhibitory and excitatory synapses. We find that for some network parameters, it is possible to observe spikes or bursts synchronization. We use the mean order parameter ($\bar{R}$) and the mean coefficient of variation ($\bar{\text{CV}}$) as diagnostic tools to identify synchronization and spikes or bursts patterns, respectively. We also calculate $\bar{F}$ and ${\bar{I}}_{\text{s}}$ to analyse how they are related to synchronous behavior.

In order to explore the effects of different delayed conductances on the neuronal synchronization, in the section 3, we consider delay in both inhibitory and excitatory conductances. When all neurons are spiking (weak coupling), the delays induce synchronization domains in the parameter space *d*_inh_ × *d*_exc_. Inside the parameter domains with synchronized neurons, we observe separated parameter subdomains representing neurons with higher and lower values of the mean firing frequency $\bar{F}$ (Hz), as well as different values of the mean synaptic input ${\bar{I}}_{\text{s}}$ (pA). For the neuronal network with strong coupling, we do not find domains with behavior similar to weak coupling in the parameter space *d*_inh_ × *d*_exc_. However, we see synchronous and desynchronous activities with either spike and burst activities. We also observe a range of high values of $\bar{F}$ and ${\bar{I}}_{\text{s}}$ when only inhibitory delayed conductance is increased (*d*_exc_ ≈ 0), responsible for turning desynchronous spikes into synchronous burst patterns. For *d*_exc_ ≈ *d*_inh_ and strong coupling, we also observed desynchronous spike activities. Desynchronous spike activities can be associated with lower mean firing frequency and synaptic currents for strong coupling.

For weak coupling, the size of the region with synchronized behavior in *d*_inh_ × *d*_exc_ decreases when the number of connections is decreased. In this situation, we observe that the size of the small regions can be increased by increasing *g*_exc_. In addition, for strong coupling and decreasing the number of connections, there is no burst activity and we verify the existence of synchronized and desynchronized spiking patterns, as shown for weak coupling and no sparse connectivity. Therefore, the connectivity and the synaptic conductance play an important role in the synchronization.

In conclusion, we verify that the delay in the conductances plays a crucial role in the behavior of the neurons in the neuronal network. For weak coupling, we uncover that not only the synchronous behavior, but also the mean firing frequency and the mean synaptic input depend on the delayed inhibitory and excitatory conductances. We identify which range of synaptic current allow the neuronal network to achieve and maintain synchronous activities. In the region with desynchronized activities, excitatory and inhibitory currents arrive in different times, consequently, high synchronization does not appear. For strong coupling, we see that also spike and burst patterns depend on the delayed conductances. The domain with synchronous pattern is characterized by having different delays in the inhibitory and excitatory conductances. Considering *d*_exc_ ≈ *d*_inh_, we observe desynchronous spikes activities for both weak and strong coupling. In addition, our results demonstrate that not only intensity of synaptic conductance, but also a short delay in the inhibitory conductance are relevant to avoid abnormal neuronal synchronization.

Our results can be useful to clarify how synchronous and desynchronous activities are reached in a context of neuronal population with delayed conductance. In future works, we plan to analyse the influence of the connection probability between excitatory and inhibitory neurons in the neuronal synchronization, as well as the appearance of clusters synchronization.

## Data Availability Statement

The raw data supporting the conclusions of this article will be made available by the authors, without undue reservation.

## Author Contributions

PP, FB, KI, EL, and MH designed the work, developed the theory, and performed the numerical simulations. AB wrote the manuscript with support from MB, IC, JS, and JK. The authors revised the manuscript several times and gave promising suggestions. All authors also contributed to manuscript revision, read, and approved the submitted version.

## Conflict of Interest

The authors declare that the research was conducted in the absence of any commercial or financial relationships that could be construed as a potential conflict of interest.
